# Micronuclei in genotoxicity assessment: from genetics to epigenetics and beyond

**DOI:** 10.3389/fgene.2013.00131

**Published:** 2013-07-11

**Authors:** Lidiya Luzhna, Palak Kathiria, Olga Kovalchuk

**Affiliations:** Department of Biological Sciences, University of LethbridgeLethbridge, AB, Canada

**Keywords:** micronuclei, chromosome-defective, epigenetics, genotoxicity, DNA methylation, histones, small RNAs

## Abstract

Micronuclei (MN) are extra-nuclear bodies that contain damaged chromosome fragments and/or whole chromosomes that were not incorporated into the nucleus after cell division. MN can be induced by defects in the cell repair machinery and accumulation of DNA damages and chromosomal aberrations. A variety of genotoxic agents may induce MN formation leading to cell death, genomic instability, or cancer development. In this review, the genetic and epigenetic mechanisms of MN formation after various clastogenic and aneugenic effects on cell division and cell cycle are described. The knowledge accumulated in literature on cytotoxicity of various genotoxins is precisely reflected and individual sensitivity to MN formation due to single gene polymorphisms is discussed. The importance of rapid MN scoring with respect to the cytokinesis-block micronucleus assay is also evaluated.

## INTRODUCTION

Micronuclei (MN), also known as Howell–Jolly bodies, were first identified at the end of nineteenth century in red cell precursors by William Howell, an American, and Justin Jolly, a Frenchman (Sears and Udden, 2011). At that time, Howell–Jolly bodies were described as remnants of nuclei of red blood cells circulating in organs with pathological features (Sears and Udden, 2011). The significance of MN was evaluated in the mid-twentieth century. Dawson and Bury (1961) found MN in red cells within the bone marrow during different pathological states. The authors also mentioned that the formation of Howell–Jolly bodies was paralleled with folic acid and vitamin B deficiency (Dawson and Bury, 1961). Not so long after, MN were described in other cells, mainly lymphocytes. Irradiation of lymphocytes *in vitro* caused a linear relationship between the dose and micronucleus induction. In the same study, the micronucleus technique was proposed as a reliable method for measuring chromosomal damages caused by cytotoxic agents “*in vivo*” (Fenech and Morley, 1985). Scoring of MN in cytokinesis-blocked binucleated cells after treatment with cytochalasin-B (Cyt-B) was postulated as a procedure of choice (Fenech and Morley, 1985).

Because the incidence of MN in peripheral blood erythrocytes of splenectomized patients rose after chemotherapy, scoring of MN in such cells was proposed in order to monitor clastogenics exposure in individuals (Schlegel et al., 1986). The belief that MN were sensitive biomarkers of genotoxicity was confirmed by development of recommended protocols, scoring methods, dose levels, choice of species, sex differences, *in vivo* and cell culture assays, and statistical considerations (Heddle et al., 1983).

The importance of scoring MN produced by the action of genotoxic agents was emphasized by many cytogeneticists. A simple Giemsa stain was initially used for MN staining followed by slide scoring (Countryman and Heddle, 1976). Later, a cytokinesis-block micronucleus (CBMN) method was reported, where Cyt-B, an inhibitor of the spindle assembly, was used to prevent cytoplasmic division after nuclear division had occurred (Fenech and Morley, 1986). The CBMN assay was proven to be more efficient in studying X-ray-induced chromosomal aberrations than other methods, including incorporating of bromodeoxyuridine (Ramalho et al., 1988). It was solely used for the assessment of chromosomal loss, breakage, and associated apoptosis and necrosis induced by different mutagens, for instance, hydrogen peroxide (Fenech et al., 1999). The main advantage of the test is its ability to differentiate MN formed as a result of clastogenic or/and aneugenic treatment. Blocking dividing cells at the binucleated stage, makes it possible to recognize chromosomal loss, chromosomal breakage, and nucleoplasmic bridges (NPBs). The latter disappear after cytokinesis if the cell is allowed to divide (Thomas et al., 2003).

The CBMN method allows accumulating virtually all cells at the binucleate stage regardless of their division kinetics, thus making the test highly sensitive. In his review, Fenech (2000) precisely described the *in vitro* technique, including detailed protocols and criteria for scoring MN. The test is also used for scoring MN in cultured lymphocytes from subjects previously exposed to genotoxins, however, it is unclear if such MN originated *in vivo* or *ex vivo* during cell culture. Therefore, Speit et al. (2011) proposed an additional scoring of MN, 24 h after starting cell culture when lymphocytes are still mononuclear.

Recognizing centromere and kinetochore sites in MN may further increase the specificity of the technique. Lagging chromosomes can be recognized by anti-kinetochore antibodies in CREST treatment (scleroderma Calcinosis, Raynaud’s phenomenon, Esophageal dysmotility, Sclerodactyly, and Telangiectasia). Alternatively, centromeric regions can be detected by FISH (fluorescence *in situ* hybridization; Mateuca et al., 2006). Centromere and kinetochore negative MN are considered to contain acentric fragments. CREST, FISH, and chromosome-specific probes allow detecting the non-random, but rather prevalent existence of specific chromosomes and fragments in MN. For instance, it was shown, that the X chromosome tends to lag behind in anaphase and is more often micronucleated than autosomes. Among autosomes, the most prevalent in MN are chromosomes 9, 1, and 16 due to the breakage in heterochromatic sites in these chromosomes (Norppa and Falck, 2003).

Today, numerous studies include micronucleus scoring for measuring DNA lesions and genotoxicity of almost any possible chemical and radioactive compound (Lau et al., 2009; Watters et al., 2009; Cveticanin et al., 2010; Lal and Ames, 2011). The results obtained by such methods are comparable with those of H2AX phosphorylation and comet assays. For instance, γ-H2AX phosphorylation in immortalized mouse embryonic fibroblasts treated with etoposide (an inhibitor topoisomerase II), methyl methanesulfonate (MMS; an alkylating agent) and bleomycin (a DNA damage agent) was increased in the dose–response relationship. The Comet assay showed similar trend during this study. The same increase in micronucleus events was also observed during all genotoxic treatments (Watters et al., 2009). Micronucleus frequency was shown to be correlated with DNA double-strand breaks (DSBs) and DNA recombination events in hematopoietic tissues of fetal mice after *in utero* exposure to benzene. Benzene is known to be a human leukomogen and such events may be the primary steps for the development of childhood leukemias (Lau et al., 2009). The influence of the micronutrient deficiency on MN formation and the initiation of hematological diseases was also shown. Therefore, understanding the link between the micronutrient status and MN frequency may be useful for the future development of novel therapeutic approaches (Lal and Ames, 2011).

It is not surprising that the micronucleus assay is also used for genotoxicity screening of new and promising applications in biology, medicine, and nanotechnology. For instance, genotoxic properties of carbon nanotubes which are used in electrical circuits, paper batteries, solar panels, as well as in medicine for cancer treatment can induce micronucleus formation and DNA DSBs in lymphocytes (Cveticanin et al., 2010). MN might induce DNA damage response (DDR). Terradas et al. (2009) described the local induction of the DDR in MN displaying γ-H2AX foci after γ-ray treatment. The existence of DSBs in MN is not surprising as MN are the products of DNA damage, but the initiation of DDR is controversial as MN do not contain DNA repair machinery. According to Terradas et al. (2012), the micronuclear envelope structure determines whether recruited repair factors can reach MN chromatin.

## MICRONUCLEI FORMATION

**FIGURE 1:**
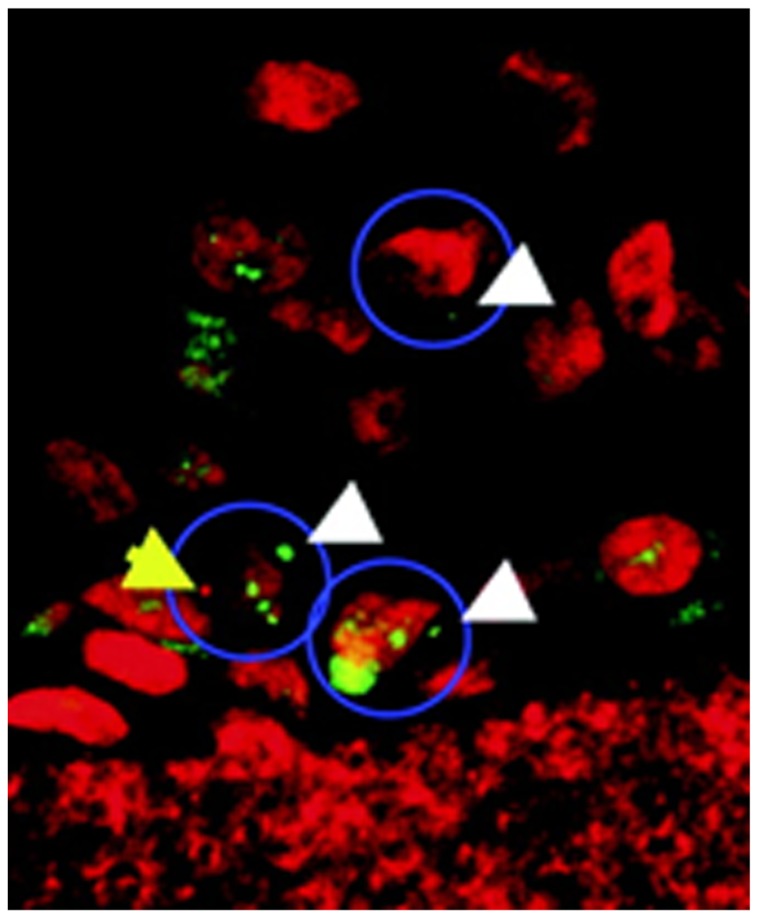
**Image of micronucleated cells in the artificial human 3D Air-100 epithelial tissue.** Nuclei and micronuclei are stained with propidium iodide (red). Micronuclei positive for γ-H2AX present green. Blue outline, micronucleated cells. White arrows, micronuclei positive for γ-H2AX; yellow arrow, a micronucleus without γ-H2AX staining. Adopted with permission from Sedelnikova et al. (2007).

**FIGURE 2:**
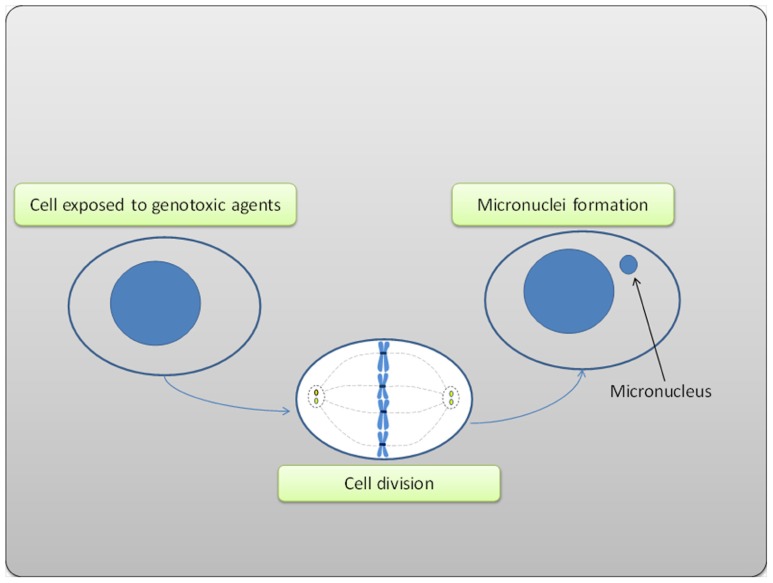
**Micronucleus formation in the cell exposed to genotoxic agent**.

Micronuclei are tiny extra-nuclear bodies originating from acentric chromatid/chromosome fragments or whole chromatids/chromosomes that lag behind at the anaphase of dividing cells and are not included in the main nucleus during telophase (**Figures 1 and 2**). Instead, they are enwrapped by the nuclear membrane and resemble the structure of the daughter nucleus, although being way smaller in size (Sedelnikova et al., 2007; Fenech et al., 2011). Acentric chromatid/chromosome fragments usually originate after extensive DNA damage such as DSBs that if misrepaired result in asymmetrical chromosome rearrangements and exchanges. Whole chromatids or chromosomes in MN are formed due to deficiencies in chromosome segregation during anaphase usually caused by mitotic spindle failure, kinetochore damage, centromeric DNA hypomethylation, and defects in the cell cycle control system (Mateuca et al., 2006).

**FIGURE 3:**
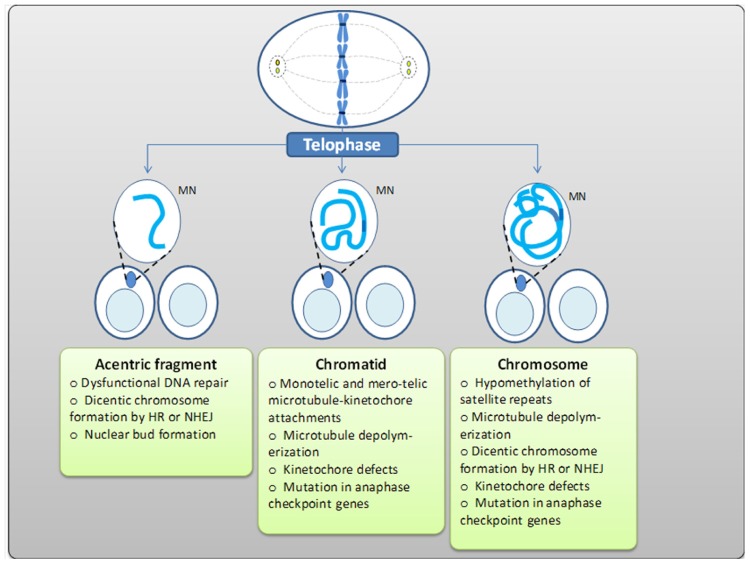
**Mechanisms of formation of MN containing acentric fragments, chromatids, and chromosomes as a result of DSBs, due to an unstable microtubule–kinetochore attachment, tubulin depolymerization, or kinetochore loss, breakage-fusion-bridge cycles, and the elimination of amplified genes through nuclear budding**.

To form an acentric fragment, DNA DSBs should either occur in one sister chromatid or extend to the whole anaphase chromosome (**Figure 3**). This happens only if the level of DSBs exceeds the repair capacity of dividing cells, which is mainly due to either the misrepair of DSBs by the dysfunctional homologous recombination (HR; O’Donovan and Livingston, 2010) or defects in enzymes of the non-homologous end-joining (NHEJ) pathway (Hartlerode and Scully, 2009). The formation of DNA DSBs and MN is often the result of simultaneous excision repair of damages and wrong base incorporation. A failure of the appropriate gap-filling event leads to DSB (Dianov et al., 1991).

**FIGURE 4:**
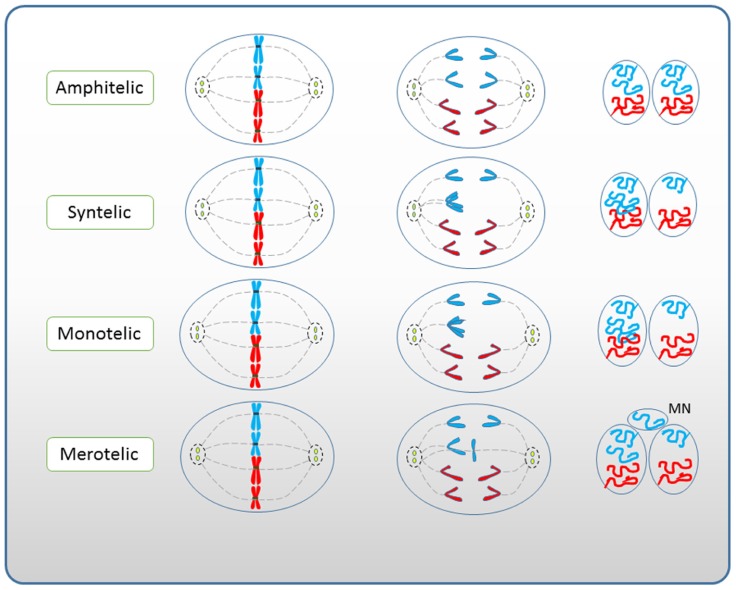
**Role of chromosome mis-segregation in micronuclei formation (modified after Cimini and Degrassi, 2005 with permission)**.

Malsegregation of sister chromatids usually happens due to the absence or inappropriate attachment of spindle microtubules to chromosome kinetochores (Cimini and Degrassi, 2005). Stable amphitelic microtubule attachments generate tension at kinetochores, locking the correct chromatid orientation in place. Unstable microtubule–kinetochore attachments such as syntelic (both sister chromatids are attached to the same spindle pole), monotelic (only one kinetochore is attached leaving the second sister chromatid unattached), or merotelic (one kinetochore is attached to both spindle poles) do not result in significant tension, thus making the bond sensitive to dissociation (**Figure 3**). If not corrected, such attachments lead to inappropriate segregation and chromosome loss, thus resulting in aneuploidy and micronucleus formation, respectively (**Figure 4**).

According to Fenech et al. (2011), the main mechanism of MN formation originated from chromosome malsegregation is hypomethylation of centromeric and paracentromeric regions – satellite repeats. Usually, satellites are hypermethylated, and loss of methylation elongates repeat regions decreasing the tension in kinetochores and thus creating wrong connections between microtubules of the mitotic spindle and chromosomes (Fenech et al., 2011). Sometimes, chromatids/chromosomes are unable to segregate as the mitotic spindle cannot pull them apart due to tubulin depolymerization. The absence of kinetochore or centromeric defects also lags chromosomes behind at telophase (**Figure 3**).

Nucleoplasmic bridges and nuclear buds (NBUDs) are similar to MN (Fenech et al., 2011). NPBs originate from dicentric chromosomes – ones that have two centromeric regions. Dicentrics are products of either HR between complementary DNA sequences of different chromosomes or NHEJ between two chromosomes that suffer from DNA DSBs (Obe et al., 2002). Similarly to the latter, NHEJ can be recruited to improperly encapsulated telomeres during their shortening. If a 3′ single-stranded overhang at the telomere is not properly capped, it is recognized as a broken DNA molecule which should be fused by NHEJ mechanism (Tarsounas and West, 2005; Longhese, 2008).

Having two centromeres, a dicentric chromosome may attach to two opposite spindle bodies which pull chromatids in the opposite directions. In the absence of breakage, the nuclear membrane surrounds both nuclei forming NPBs between them. Eventually, NPB is broken during cytokinesis, resulting in a micronucleus formation (**Figure 3**; Fenech et al., 2011). Misrepair of two chromosomal breaks causes the formation of both dicentric and acentric fragments. The latter will form MN of its own (**Figure 3**). Telomere fusion results in NPB that after breakage will accompany one of the daughter nuclei in the form of MN. MN and NPBs formed after telomere fusion contain telomeric sequences which can be recognized by specific probes that hybridize to subtelomeric regions (Boukamp et al., 2005). In contrast, MN and NPBs originated from DSBs misrepair are telomere negative (Fenech, 2007).

Anaphase bridges are initial events in breakage-fusion-bridge (BFB) cycles which are the features of chromosomal instability. The uneven breakage of NPBs leads to the formation of two daughter nuclei, one of which gained extra genetic information, whereas the second one lost an equal amount of genetic information. Such broken chromosomes usually do not contain telomeric zones and, therefore, can fuse with their replica during the next mitotic event, repeating the cycle for the next couple rounds. BFB cycles lead to the amplification of genes near the break point which are eventually looped out of the abnormal chromosome, thus forming the so-called double minutes (DMs). DM chromosomes are selectively located at the periphery of the nucleus and are eliminated from the nucleus by nuclear budding during S phase (Shimizu et al., 1998). A NBUD is virtually the same as a micronucleus, except for its closer location and connection to the nucleus through a narrow cytoplasmic passage. NBUD also contains interstitial or terminal fragments without centromeric or telomeric regions, whereas MN is formed from a lagging chromosome (Dutra et al., 2010).

While briefly summarizing the mechanisms of MN formation, it is important to emphasize that MN containing acentric chromatids or chromosomes are the result of unrepaired or misrepaired DNA breaks, whereas MN with whole chromatids/chromosomes are formed due to (a) hypomethylation of satellite centromeric/paracentromeric sequences, (b) kinetochore defects, (c) dysfunctional spindle, and (d) mutations in anaphase checkpoint genes (**Figure 3**). NPBs originate from misrepaired DNA breaks, telomere end fusion, or failure of sister chromatids to separate due to the lack of decatenation. Last but not least, nuclear budding occurs as a result of either elimination of amplified DNA resulted through BFB cycles or specific elimination of excess chromosomes in polyploidy cells (Wang et al., 2004).

## ROLE OF EPIGENETICS IN MICRONUCLEI FORMATION

Understanding of the mechanisms of MN formation induced by genotoxic agents is of a great significance for both the detection of diseases such as cancer and their treatment. The manipulation of such mechanisms may be beneficial for both the prevention of MN formation and development of diseases and the induction of MN for therapeutic purposes. In fact, it is a matter of choosing the right target in the process of MN formation.

Epigenetics has recently become a very promising target for manipulation in molecular biology because of the growing evidence of its involvement in chromatin status regulation, gene expression; and both epigenetics and genetics have an equal influence on the development of genomic instability and cancer (Aypar et al., 2011). The greatest potential of epigenetic alterations is their reversible nature in contrast to mutations which made epigenetics so attractive for therapeutic research.

The initial understanding of epigenetics proposed by Waddington reflected a model of gene interaction with their surroundings. Depending on a gene and its surrounding, such interaction produced a specific phenotype. Nowadays, epigenetics is rather defined as a memory of stable changes in gene expression without changes in gene sequence, and such memory can be passed on to progeny (Jiang et al., 2008). Such memory explains differences between genetically identical cells in a multicellular organism. Thus, gene expression in functionally different cells is epigenetically regulated. The ability of cells to change gene expression without altering gene sequence not only allows for maintaining tissue identity but also gives a possibility for the adaptation to a changing environment, should such changes occur (Jirtle and Skinner, 2007). Because transcription requires the cooperative effort of chromatin, the protein complexes that modify chromatin structure and transcription factors, the objective of epigenetics is to find out how both the genetic code in the DNA sequence and the way that the DNA is packaged control gene expression (Bock and Lengauer, 2008). The contribution of epigenetic alterations to MN formation is now clearly evident, and research on the epigenetic mechanisms involved in MN is growing. Epigenetic regulation includes at least four outlined mechanisms: DNA methylation, histone modifications, chromatin remodeling, and non-coding RNA expression (Bonasio et al., 2010; Gibney and Nolan, 2010). DNA methylation was discovered first and, therefore, is the most extensively studied. It is the only epigenetic mechanism that directly targets DNA. A methyl group replaces a hydrogen atom in the cytosine base of DNA, thus creating a new covalent bond. Such modification happens predominantly in cytosine-phosphate-guanine (CpG)-dinucleotides (Bird, 2009). The addition of a methyl group does not affect the transcription of cytosine, but it alters chromatin in a way such that to interfere with and reduce DNA-binding capacities of transcription factors (Weber and Schubeler, 2007). Methyl-CpG-binding proteins (MBPs) recruit transcriptional suppressors to modify chromatin (Fujita et al., 2003; Kondo et al., 2005). Enzymes that methylate DNA are DNA methyltransferases: DNMT1, DNMT2, DNMT3a, and DNMT3b. DNMT1 can maintain a DNA methylation pattern by reading and faithfully copying it from an old DNA strand to a newly synthesized strand during replication. DNMT3a and b target unmethylated CpG sites for *de novo* methylation in embryonic stem cells and cancer cells (Okano et al., 1999). Such methylation activity is important for the establishment of parental imprints (Kato et al., 2007). DNMT2 has been shown to methylate tRNA (Goll et al., 2006) in addition to a weak methyltransferase activity *in vitro* (Hermann et al., 2003).

The role of DNA methylation is crucial for normal development, proliferation, and genome stability. The distribution of CpG-dinucleotides is not random in the genome. Most of CpG sites are clustered in promoter areas of genes creating so-called CpG islands (Bird, 2009). Usually, promoters of tumor suppressor genes are hypomethylated to allow their expression for normal functioning of cells (Herman and Baylin, 2003), whereas oncogenes and some repeat elements are silenced through hypermethylation, thus maintaining genome integrity (Huang et al., 2004). Reanimated transposons can lead to translocations, gene disruption, and chromosomal instability (Bestor, 2005). X chromosome inactivation is also a result of hypermethylation (Reik and Lewis, 2005). Centromeric regions of chromosomes are heterochromatic and lay within tandemly repeated DNA. Constitutive heterochromatin of centromeres is epigenetically silenced by histone methylation (H3K9Me3 and H3K27Me) and DNA hypermethylation, thus enabling a low frequency of recombination and the repression of transcription (Peters et al., 2003; Grunau et al., 2006). However, undermethylation of repeated DNA sequences and satellite DNA in the centromeric and pericentromeric regions of chromosomes is highly linked to karyotypic instability found in a variety of cancers (Ehrlich, 2002). The possibility exists that DNA hypomethylation in the centromeric region may modify a platform for the correct kinetochore orientation and attachment to the spindle, resulting in improper chromosome segregation and MN formation. Such hypothesis is mainly supported by ongoing experiments involving DNA methylation activators/inhibitors which affect MN formation. For instance, treatment of human fibroblasts with *S*-adenosyl-methionine (SAM) reduces the frequency of MN caused by sodium arsenite (NaAsO_2_; Ramirez et al., 2007). The genotoxicity of arsenic is characterized by the generation of free radicals, changes in DNA methylation patterns, the inhibition of DNA repair and formation of MN. Interestingly, in this study, SAM decreased arsenic-induced MN containing only whole chromosomes but not fragments. This means that SAM may prevent chromosome loss and aneuploidy, but it is not capable of reducing chromosome breaks and clastogenicity. According to the authors, NaAsO2 is a demethylating agent which may possibly disturb chromatin condensation in the pericentromeric region affecting the correct kinetochore orientation and attachment to the spindle; and SAM, as a methyl group donor, may correct chromosome alignment and segregation (Ramirez et al., 2007). Another possible explanation relied on SAM as a potent antioxidant agent which may prevent the oxidative stress caused by arsenic compounds, but in such case, a decrease in DNA DSBs would be expected, yet it did not happen. The data of the study supported the idea that SAM protects chromosome segregation by the restoration of DNA methylation status, preventing MN induction (Ramirez et al., 2007). In one of the earlier studies on lymphocyte cultures, the authors demonstrated that 5-azacytidine induced a significant undercondensation of heterochromatic regions of chromosomes 1, 9, 15, 16, and Y which correlated with an increase in MN formation (Guttenbach and Schmid, 1994). The study showed that the chromosome that was most frequently eliminated in MN was chromosome 1 (41%). Undercondensation of heterochromatin in chromosome 1 was the most frequent event (52% of metaphases). In contrast, chromosomes 11, 17, and X contained 5-azacytidine-resistant heterochromatin and were not included in MN formation (Guttenbach and Schmid, 1994). A year later, Stopper et al. (1995) demonstrated MN induction by four analogs of cytidine: 5-fluoro-2′-deoxycytidine, 5,6-dihydro-5-azacytidine, 5-azacytidine and 6-azacytidine. All four analog induced MN, but the lack of kinetochore staining in most of MN indicated that the compounds were clastogenic (Stopper et al., 1995). 5-Azacytidine functions as an inhibitor of DNMTs which recognizes the analog as the natural substrate in the methylation reaction, thus becoming trapped and degraded (Stresemann et al., 2006). A recent study indicates that global DNA hypomethylation correlates with MN formation, increased ploidy and DNA damage in equine sarcoid-derived fibroblasts affected by bovine papillomavirus (BPV-1; Potocki et al., 2012). A similar correlation between MN induction and DNA hypomethylation was shown in radiation-induced bystander cells. An increase in the level of MN, DNA DSBs, and apoptosis was parallel to the loss of nuclear DNA methylation in bystander human cells after microbeam radiation (Sedelnikova et al., 2007).

Furthermore, recent studies show association between folate levels and MN. Folate is an important B group vitamin that partakes in a complex homocysteine cycle which yields SAM – a key methyl donor for DNA methyltransferases (Fuso, 2013). Therefore, folate is crucially important for DNA methylation. Recent studies have shown that folate deficiency is associated with genomic damage and formation of MN and other nuclear abnormalities in human lymphocytes (Bull et al., 2012; Lu et al., 2012). Furthermore, folate supplementation led to a pronounced reduction in DNA damage and MN formation (Lazalde-Ramos et al., 2012). These data provide additional support to the epigenetic mechanisms of MN formation.

Based on all the aforementioned facts and lines of evidence, we may conclude that MN formation is induced epigenetically mainly through the loss of DNA methylation. Specifically, hypomethylation of heterochromatin in the pericentromeric regions is associated with chromatin decondensation, which leads to improper chromosome segregation and exclusion into MN; whereas global hypomethylation is associated with more relaxed chromatin, increased gene expression, elevated DNA damage, and chromosomal breaks which form MN with acentric chromosome fragments. Therefore, hypomethylation is related to both aneugenic and clastogenic mechanisms of MN formation. Nevertheless, such hypothesis needs further investigation and more experimental evidences.

DNA in eukaryotes is not naked; it is combined with histone proteins into chromatin. A relaxed chromatin state is implicated in numerous biological processes such as replication, transcription, and repair. Chromatin condensation can become an obstacle for these processes. Dynamic changes in chromatin structure provide balanced cellular activities such as proliferation, cell cycle progression, apoptosis, etc. Uncontrolled chromatin remodeling can result in dysregulated gene expression and cancer initiation. The unit of chromatin, termed the nucleosome, consists of four histones: H2A, H2B, H3, and H4. DNA is wrapped around histones, and a linker histone H1 stabilizes the octamer structure (Ma et al., 2010). The amino-terminal tails of core histones (25–40 residues) are not wrapped around DNA but extend into surrounding space and, therefore, can be targeted by specific histone modificators. The main histone modification events known to date are: methylation, acetylation, phosphorylation, and ubiquitination. Histone modifications do not cause changes in the DNA sequence, but they lead to changes in the chromatin state which alters gene expression (Ma et al., 2010). Histone tails are rich in lysine amino acid residues which provide a positive charge to histones. Positively charged histones interact with negatively charged DNA, and a tight connection between DNA and histones is achieved. The presence of acetylated lysins in histone tails lowers the positive charge leading to a relaxed chromatin state. An opposite event, deacetylation, represses gene expression (Jenuwein and Allis, 2001). Methylation events in lysine residues cause different chromatin states, depending on the position at which histone methylation occurs.

Heterochromatin structure is determined by both CpG methylation and methylation of histone tails. High levels of methylation of histone 3 at lysine residue 9 (H3-K4) and low levels of methylation at H3-K4 are heterochromatin-specific methylation marks at the pericentromeric DNA that are controlled by Lsh (lymphoid-specific helicase), a member of the SNF2 chromatin remodeling family. Loss of Lsh results in di- and tri-methylation of H3-K4 in repetitive sequences (Yan et al., 2003). In addition, Lsh-deficient fibroblasts tend to form multipolar spindles, accumulate high centrosome numbers and last but not least display an increase in MN formation (Fan et al., 2003). Moreover, the most prevalent type of acetylation of histones H3 and H4 in embrional fibroblast cells derived from Lsh^-^^/^^-^ embryos was found in all repetitive elements such as major and minor satellite sequences and IAP retroviral-derived sequences (Huang et al., 2004; Muegge, 2005). These studies indicate the importance of histone modifications and chromatin remodeling in chromosome instability and possibly in the formation of MN. Additionally, the two key pioneering studies have shown the crucial role of altered histone histone acetylation in MN formation (Qiu et al., 2000; Fujita et al., 2007).

Currently, only a few studies indicate that microRNAs (miRNAs) are involved in the induction of MN. miRNAs are known to regulate gene silencing in mammals, fish, frogs, insects, worms, flowers, and viruses. Approximately 2–3% of the human genome encode for miRNAs (Alvarez-Garcia and Miska, 2005). miRNAs are important for cellular proliferation, apoptosis, differentiation, tissue and organ developing. It is now well-known that aberrant expression of miRNAs is associated with cancer development and progression. miRNA genes are encoded in cellular DNA and transcribed by RNA polymerase II into large RNA precursors called pri-miRNAs (500–3000 bases) that are 5′ 7-methylguanosine-capped and polyadenylated (Liu et al., 2008). In the nucleus, pri-miRNAs are microprocessed by Drosha and Pasha (also known as DiGeorge-syndrome critical region protein 8 – DGCR8). The products of Drosha and Pasha are ~70-nucleotide pre-miRNAs fold into stem-loop structures with a 2-nt 3′ overhang (Lee et al., 2003; Landthaler et al., 2004). Pre-miRNAs are exported from the nucleus to the cytoplasm by RAN GTP-dependent exportin 5. In the cytoplasm, pre-miRNAs undergo further processing by another RNAse III-family/type endonuclease called Dicer (Lee et al., 2002). Dicer excises an imperfect miRNA duplex from the pre-miRNA hairpin creating double-stranded RNA of ~22 nucleotides in length. Such miRNA duplex can be incorporated into and form the RNA-induced silencing complex (RISC). Dicer along with the trans-activation response RNA-binding protein (TRBP), PACT, nuclease Tudor-SN and Argonaute (AGO) proteins contribute to the formation of RISC (Kim et al., 2009). In the RISC complex, the miRNA duplex is unwounded by specific helicases to form a single-stranded mature miRNA that is capable to negatively regulate its target mRNAs. The second strand miRNA is degraded. MiRNAs with nearly perfect complementarity to the mRNA sequences induce the RNA-mediated interference (RNAi) pathway. miRISC binds within the open reading frame (ORF) and its specific ribonucleases, mainly Argonaute 2, and cause cleavage of mRNA, thus resulting in mRNA degradation. This mechanism is believed to be predominant in plants, but it was also proven to happen in mammals. However, most animal mRNAs are thought to be down-regulated rather than cleaved. By this mechanism, miRNAs bind to the imperfect complementary sequences within the 3′ untranslated regions (UTRs) of target mRNAs and repress mRNA gene expression (Stark et al., 2005). In such a way, the levels of proteins are reduced, but the levels of mRNAs remained stable. The RISC complex is known to bind active chromatin sites in yeast and plants, thus causing histone methylation and transcriptional inactivation.

A recent study of Aypar et al. (2011) showed an immediate induction of MN following radiation exposure which was paralleled with alterations in DNA methylation and miRNA expression. High- and low-linear energy transfer (LET) radiation resulted in hypomethylation of repeat elements LINE-1 and Alu and the differential expression of six (after low LET) and three (after high LET) miRNAs in human–hamster hybrid cells GM10115. The identified targets for those miRNAs were involved in the five major pathways: DNA repair, cell cycle checkpoint, apoptosis, chromatin remodeling, and DNA methylation. The authors stated that such epigenetic changes may contribute to radiation-induced genomic instability. Because MN formation is one of the hallmarks of genomic instability, miRNA mis-expression may be one of the mechanisms of MN induction – the hypothesis is waiting to be tested.

A major class of siRNAs is encoded by repetitive sequences within heterochromatic regions of centromeres in plants, fungi, and animals (Ambros et al., 2003; Xie et al., 2004). Such repeat-associated siRNAs (rasiRNAs) are larger than miRNAs, and their presence is correlated with a repressed chromatin state (RNA-induced transcriptional silencing, RITS) of those very regions from which RNAs are transcribed (Sontheimer and Carthew, 2005). Alterations in the RITS complex of *S. pombe* led to aberrations in mitosis and meiosis, such as high rates of non-disjunction and lagging chromosomes in mitosis and mis-segregation during meiosis (Hall et al., 2003). The possibility exists that mammalian rasiRNAs function through a similar pathway for the regulation of heterochromatin (Holmes and Cohen, 2007).

**FIGURE 5:**
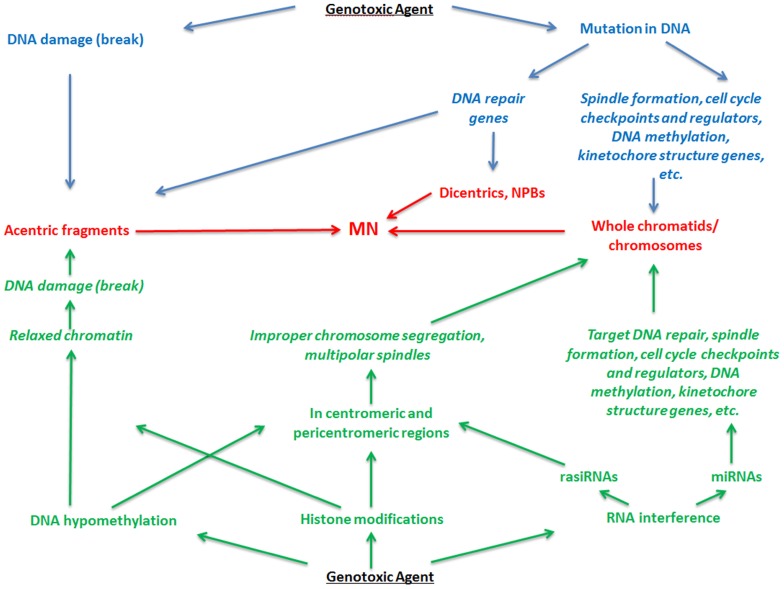
**Role of the genetic and epigenetic factors in the formation of micronuclei.** MN and its components are reflected in red, genetic factors are reflected in blue, and epigenetic factors are reflected in green.

Overall, centromeres play a key role in cell division being the sites of kinetochore assembly necessary for capturing microtubules and pulling the sister chromatids to opposite poles of the spindle. They are rich in repetitive DNA, heterochromatic, and subject to epigenetic modifications. The coordinated epigenetic silencing of centromeric heterochromatin is essential for maintaining correct chromosome segregation. Therefore, epigenetic alterations that lead to a more relaxed chromatin state in centromeres may directly result in lagging chromosomes and MN formation. Given a significant role of epigenetics in centromere behavior, it is still not clearly revealed how exactly epigenetic modifications are associated with MN formation. Although the importance of DNA methylation and chromatin remodeling in centromere behavior has been shown, the role of RNAi pathways remain obscure. The summarized roles of both genetic and epigenetic factors in MN formation are outlined in **Figure 5**.

## THE FATE OF MICRONUCLEI IN CELLS

Analysis of the fate of MN in the cells has recently regained a lot of interest. The recent study by Utani et al. (2010) suggested that MN formed after mitosis were stably maintained in the cells for up to one cell cycle. Furthermore, mitotic division of cells with MN led to formation of daughter cells either with or without MN.

The ability of MN DNA to replicate itself remains obscure, but some suggestions have been made that MN replication depends on MN nature, and if it happens, usually it occurs at the same time as main nucleus replication (Obe et al., 1975). Similarly, MN transcription events depend mainly on MN structure. MN containing whole chromosomes showed active transcription (Labidi et al., 1987), whereas acentric fragments were not able to synthesize RNA (Hoffelder et al., 2004), unless they represented transcriptionally competent DMs (Utani et al., 2007). It should be emphasized here that any possible transcriptional activity in MN depends on nuclear envelope integrity and the presence of nuclear pore complexes (NPCs; Geraud et al., 1989; Sukegawa and Blobel, 1993).

The DDR in MN involves the formation of ionizing radiation-induced foci (IRIF), similar to ones in the main nucleus. γ-H2AX foci were co-localized with ATM and MDC1 proteins (Medvedeva et al., 2007). The observation of HR protein Rad51 and ss-DNA-binding protein RPA in radiation-induced MN leads to the suggestion that DDR proteins are either randomly entrapped in MN together with damaged DNA or are recruited to MN that contain damages (Terradas et al., 2010). Similarly, TP53 accumulation in MN was observed to trigger DDR (Granetto et al., 1996). Cells treated with colchicine, vinblastine, bleomycin, and arsenic showed a significant induction of MN and p53 (Salazar et al., 2009). By analogy with DNA replication in MN, DNA repair may also be compromised by micronuclear envelope trafficking abilities (Labidi et al., 1987). Multiple studies showed the existence of apoptotic-like DNA degradation in MN that were unable to repair DSBs (Terradas et al., 2009). Such MNi are expulsed from the cell and are lost forever. The effect of MN expulsion on a cell can be dual. If destroyed MNi carried extra chromosomes in the cell, then their elimination would be necessary for regaining the normal cellular status, but if the MN chromosome was complementary to the main nucleus, then the cell might lose a certain gene dosage (Terradas et al., 2010).

Some micronucleated cells originating from the loss of chromosomes can be eliminated by apoptosis. For instance, nocodazole, a microtubule inhibitor and blocker of cell cycle at M-phase, gives rise to aneuploid, polyploid, and micronucleated cells. It was observed that such MN-carrying cells were apoptotically eliminated through the activation of caspase-8, caspase-9, and effector caspase-3 (Decordier et al., 2008). Interestingly, when MCF-7 cells lacking caspase-3 were treated with nocodazole, MN induction decreased, which allowed the authors to suggest a possible role of caspase-3 in MN formation (Decordier et al., 2005). There is also data suggesting the reincorporation of MN into the main nucleus and the restoration of normal biological activity in the cell. Alternatively, retention of MN within the cell as an extra-nuclear entity is also possible (Leach and Jackson-Cook, 2004).

Micronuclei formation in humans is associated with various medical conditions. MN in spermatids may lead to infertility, while a high number of MN in lymphocytes is associated with pregnancy complications and miscarriages (Fenech, 2011). MN are one of the four main endpoints, together with chromosomal aberrations, aneuploidy, and sister chromatid exchange (SCE) in the identification of cancer initiation (Tucker and Preston, 1996; Hagmar et al., 2001). A large number of papers describe the correlation between MN and cancer development. A significant increase in MN in lymphocytes was shown in untreated cancer patients (Iarmarcovai et al., 2008b). Furthermore, healthy women with BRCA1 and BRCA2 mutations showed a higher increase in MN frequency and a higher radiation sensitivity than women without family history of breast cancer (Rothfuss et al., 2000; Trenz et al., 2003). Similar outcomes were shown in lung cancer patients with a high frequency of spontaneous MN (Guler et al., 2005), as well as in patients with pleural malignant mesothelioma (Bolognesi et al., 2002), and adenocarcinoma patients (Karaman et al., 2008). Cancer-prone patients with Bloom syndrome and ataxia telangiectasia also possess a high frequency of MN in lymphocytes (Rosin and German, 1985). Analysis of European cohorts indicates that individuals with increased MN are more likely to get cancer 12–15 years after the test was performed (Bonassi et al., 2007).

## MICRONUCLEI AND GENOTOXIC AGENTS

Over the past century, different cytogeneticists have been studying and describing the genotoxic effect of multiple exposures on cells and organisms, relating such exposures to chromosomal aberrations, genomic instability, and cancer development (Kirsch-Volders et al., 2003). The potential of ionizing radiation to directly or indirectly cause damage to DNA is a good example of such genotoxic influence. Different genotoxic agents use a variety of different mechanisms to alter DNA structure and affect nuclear integrity (**Table 1**). For instance, mitotic spindle disrupting agents cause chromosome malsegregation (Elhajouji et al., 1995), while metals have a variety of cellular targets (**Table 1**). Therefore, genotoxic agents have been classified into two classes according to the mechanism of action: clastogens which cause breaks in chromosomes, and aneugens which affect cell division and mitotic spindle apparatus, leading to aneuploidy. Accordingly, MN formed as a result of clastogenic or aneugenic treatment will differ in their content. Thus, clastogens and aneugens will form MN with acentric fragments and whole chromosomes, respectively (Terradas et al., 2010).

**Table 1:** Role of selected genotoxic agents in micronuclei formation.

Ionizing radiation	Anti-mitotic agents (vinca alkaloids)	Metals	DNA methylating agents	Anthracycline drugs
DNA and protein adducts, DNA strand breaks, crosslinks, gene mutations	Mitotic spindle disruption, chromosome malsegregation	By binding to DNA and proteins cause damage to DNA, altered gene expression, mutations, altered cell cycle, chromosome non-disjunction, cytoskeleton dysfunction	Loss of methylation in centromeric and paracentromeric regions, sister chromatid decondensation, improper chromosome segregation, chromosome breaks	DNA damages, disruption of DNA replication and DNA repair, DNA breaks
Mainly clastogenic effect, but mutations in certain cell cycle and repair genes may cause aneugenic MN	Mainly aneugenic	Both clastogenic and aneugenic effects, depending on the metal	Aneugenic, if hypomethylation in centromeric regions; clastogenic if global hypomethylation leading to DNA strand breaks	Mainly clastogenic

The role of IR in MN production is relatively well understood. The production of MN in human lymphocytes after X-ray treatment was observed at the time of the first mitosis, 48 h after the culture was started (Countryman and Heddle, 1976). The frequency of MN was much lower when the fractionated dose of X-rays was applied. Highly energetic beta particles of (90)Sr/(90)Y caused the induction of MN by an exponential quadratic model in Chinese hamster ovary (CHO) cells (Murakami et al., 2004). Similarly, X-rays and UV caused a dose-dependent MN induction with a slope factor of 1.8 and 10.3 for X-rays and UV, respectively (Fenech and Neville, 1992). Cytogenetic results of IR-exposed individuals in the Southern Ural showed a higher frequency of MN in lymphocytes and a lower adaptive response, compared to Moscow people (Akleev et al., 2002). In their study, Kamiguchi and Tateno (2002), evaluated MN as markers of chromosomal aberrations in human and hamster and mouse spermatozoa irradiated *in vitro* by five kinds of ionizing radiation (137C gamma, 60Co gamma, X-rays, 3H beta, and 252Cf neutrons). Radiosensitivity measured by the MN test was the highest in human spermatozoa, followed by hamster and mouse cells. The authors emphasized the importance of sperm chromosome analysis for cancer patients treated with radiotherapy (Kamiguchi and Tateno, 2002). Different results were described for X-irradiated human and canine lymphocytes. MN yield in canine cells after 1 and 2 Gy was about 1.3 times higher than in human cells. Such a difference may be due to a different chromosome number in dogs and humans (78 and 46 chromosomes, respectively; Catena et al., 1994).

The combination of ionizing radiation and certain chemical compounds or drugs can result in an absolutely different response compared to that caused by IR or drug alone. For instance, X-rays increase the frequency of micronucleated polychromatic erythrocytes (MNPCE) in mice in a similar way to the S-2-/3-aminopropylamino/ethyl phosphorothioic acid (WR-2721). If WR-2721 was administered before IR, the frequency of MN was reduced, showing the protective effect of the drug on IR toxicity (Mazur, 1995). In contrast, 3-aminobenzamide increased the IR-induced MN in human lymphocytes (Catena et al., 1992). Supra-additivity by 34–86%, was described for the combination of gamma-IR and ethyl methanesulfonate in mouse lymphoma cells (Stopper et al., 2000). Finally, the combination of doxorubicin and gamma-radiation increased the frequency of MN and death rates in HeLa cells; thus, it allowed authors to make a conclusion that combinational treatment increases the genotoxic effect of the either treatment given alone (Jagetia and Nayak, 2000).

Micronuclei testing is widely used for the evaluation of genotoxicity of different anti-cancer drugs. Adriamycin is an anthracycline drug with strong mutagenic properties that increases MN incidence up to 10- to 15-fold and significantly declines cell survival (Bhuyan et al., 1983; Jagetia and Nayak, 2000). Curcumin alone induces MN in PC12 cells but reduces the total frequency of MN induced by cisplatin, thus showing both genotoxic and antigenotoxic properties, depending on prescription protocols (Mendonca et al., 2009). Similarly, anti-cancer drugs, gemcitabine and topotecan, increase abnormal metaphases and the number of MN in mouse bone marrow (Aydemir and Bilaloglu, 2003). The CBMN assay showed the stimulation of DNA damages in V79 Chinese hamster cells after combinational treatment with bleomycin and DNA-PK inhibitor wortmannin (Oliveira et al., 2002).

Vindesine, an anti-mitotic vinca alkaloid, if combined with gamma-radiation, reduces survival of V79 cells by increasing the frequency of MN (Jagetia and Adiga, 2000). MN caused by breakage events were noticed in human lymphocytes treated with antineoplastic drug ASE (Andrianopoulos et al., 2000). Teniposide, an anti-tumor drug used for treatment of childhood acute lymphocytic leukemia, induced MN with a peak frequency at 16 h after treatment, which was correlated with cell survival decline (Adiga and Jagetia, 1999). An interesting genotoxic mechanism of action of natural alkaloids of pyrido-thiazolo-acridine series was observed; acridines acted through the DNA-intercalating mechanism in the dark, but DNA-adducts were formed after photo-activation (Di Giorgio et al., 2008). Last but not least, methylating agents, MMS and *N*-methyl-*N*-nitrosourea (MNU), generated a linear dose response in MN formation (Lutz et al., 2005).

While MN formation is the wanted result caused by tested anti-cancer drugs, it is a less desirable endpoint in testing of multiple drugs used to treat a variety of diseases other than cancer. Thus, MN induced by such treatments are considered to be a side effect of drug action. One example is the cytogenetic toxicity of metronidazole (a common Rosacea treatment agent) which causes MN induction in somatic cells of mature male Swiss mice and is correlated with anemia development and decreased male fertility (el-Nahas and el-Ashmawy, 2004). MN evaluation among 79 renal transplant patients showed that immunosuppressive therapy induces mutagenic side effects resulting in chronic renal failure (Rath and Oliveira-Frick, 2009). Acyclovir, the most common agent to treat cold sores, was shown to enhance radiation effects on MN formation in HeLa cells, although it does not demonstrate a possible side effect of the drug during its specific treatment (Jagetia and Aruna, 2000). An anti-leukemic drug, Myleran (R), caused MN in rat lens epithelial cells, thus proving the hypothesis about cataractogenicity of certain drugs (Odrich et al., 1988). Similarly, AIDS treatment with nucleoside analogs increased MN in bone marrow, which suggests that intrinsic genotoxic activity of nucleoside analogs should be considered during selection of drug administration for AIDS treatment (Phillips et al., 1991).

A series of studies indicate similar results in terms of genotoxic potential and MN induction for: *Strychnos pseudoquina*, a Brazilian medicinal plant with novel antiulcerogenic activity, that was shown to give rise to MN in blood cells (Santos et al., 2006), West African plant *Cryptolepis sanguinolenta* which contains an anti-malarial herbal alkaloid, cryptolepine, that induces MN in hamster fibroblast cells (Ansah et al., 2005), anti-obesity drugs sibutramine and fenproporex that induce MN in Swiss mice (da Silva et al., 2010), and some antibacterial/antiviral drugs possessing clastogenic and aneugenic properties (Abou-Eisha et al., 2004; Balakrishnan and Eastmond, 2006; Nersesyan et al., 2011).

Genotoxicity of the environment and manufactory pollution has always been an important issue (Buchet et al., 1995; Ibrulj et al., 2006). In their study, Neri et al. (2003) described the effect of various environmental mutagens on the frequency of MN in children (0–18 years). A higher sensitivity to pollution and a minor role of cigarettes smoking and lifestyle makes children a better model population for environmental cytogenetic monitoring. Common genotoxic agents, such as ionizing radiation, air pollution, and chemical drugs, cause an increase in MN frequency in children (Neri et al., 2003).

Hornhardt et al. (2006) showed that the combination of arsenic trioxide in the concentration close to that occurring in nature induces MN in human lymphoblastoid cells if combined with gamma-radiation. Similar observations were made for genotoxicity of chelate complexes of mercury (II) employed in detoxification of some polluted areas. The complex of mercury (II) with EDTA interferes with tubulin assembly in V79 cells (Stoiber et al., 2004). The insecticide lindane increases the number of MN up to fivefold in MCF-7 breast and PC-3 prostate cells 24 h post-treatment (Kalantzi et al., 2004), while four commercial pesticides used in Italian agriculture induce genotoxic damage only if used in high toxic doses. These were Citroxin (CX), Decis (DS), Tramat Combi (TC), and Lasso Micromix (LM), containing following agents – Alachlor (in LM), Clopyralid (in CX), Ethofumesate and Lenacil (in TC), and Deltamethrin (in DS; De Marco et al., 2000). In a series of studies, Dorn et al., 2008a,b, evaluated clastogenic and aneugenic effects of various anabolic steroids misused by athletes in sports. Most of these steroids induced MN in V79 cells up to 2-fold compared with controls, thus, presenting a potential genotoxic hazard (Dorn et al., 2008a,b). The potential hazards of dental adhesives interacting with pulp tissues can also be expected. Dental adhesives cause the generation of ROS contributing to MN formation up to 6-fold in V79 cells (Demirci et al., 2008). MN tests also confirm a slight genotoxic potential of the common ingredient of oxidative hair dyes, *p*-phenylenediamine (PPD), *in vitro*, but not *in vivo* (Garrigue et al., 2006).

Continuing to discuss genotoxicity, it should be mentioned, that aspartame, a low-calorie sweetener, can be mutagenic and may cause chromosome aberrations and MN (Rencuzogullari et al., 2004). On the other hand, genotoxicity of human breast milk is unexpected. Samples of human milk of healthy nursing mothers were positive in bacterial tests for mutagenicity (Ames test) and in tests for clastogenicity (MN test). Human milk extracts induced MN in MCL-5 (human lymphoblastoid cells), while cells treated with cow’s milk and corn oil were MN negative (Martin et al., 1999). In the same manner, soy isoflavones in human diet were used for studies of chromosomal genotoxicity. It was shown that isoflavone genistein caused a dose-related formation of MN in a dose range of 5–25 μM in V79 cells. Most of the MN did not contain kinetochores, indicating the clastogenic mode of action of genistein (Di Virgilio et al., 2004). A dose-dependent MN formation was discovered in ovine seminal vesicle (OSV) cell cultures treated with ochratoxin A (toxin produced by *Aspergillus* and some *Penicillium* species) found in common foods. The majority (70%) of MN was kinetochore-positive, pointing to an aneugenic effect of ochratoxin (Degen et al., 1997).

Because genotoxicity is linked to chromosome aberrations, it is expected that cigarette smoking would cause MN. Surprisingly, most studies deny the ability of smoking compounds to induce MN. In the Human MicroNucleus project, 1409 current smokers and 800 former smokers were tested for MN in lymphocytes. Both groups showed a decrease in MN frequency compared to non-smokers (Bonassi et al., 2003). Although, when tobacco-specific nitrosamine (NNK) was added to the culture of the repair-deficient fibroblasts, the frequency of MN was doubled (Pohlmann et al., 1992) suggesting that smoking could induce MN in repair-deficient cells.

Multiple studies describe kinetics of MN induction by different genotoxic agents (Przybojewska, 1992). For instance, some vinca alkaloids block cell division immediately, while vinblastine and vincristine cause a delay after exposure, although producing a higher maximal velocity (Morales-Ramirez et al., 2004). The induction of MN by colchicine also occurs rapidly; MNPCE appeared in blood stream almost at the same time as after exposure to gamma-rays (Vallarino-Kelly and Morales-Ramirez, 2001). A long latency period in MN formation was observed after methylnitrosourea, thus proving that the agent causes DNA breaks through the repair of mismatches induced during a previous division. Therefore, a relationship exists between the kinetics of MN and chromosomal break formation (Morales-Ramirez and Vallarino-Kelly, 1999).

## ANTICLASTOGENIC AGENTS AND MICRONUCLEI

Considering a growing number of genotoxic agents, anti-mutagenic properties of some compounds are of great value (Raj et al., 2001). According to Heo et al. (1992), most flavonoids possess anticlastogenic properties. The authors tested 14 flavonoids against the induction of MN by benzo[*a*]pyrene in polychromatic lymphocytes in mice. All of them showed a dose-dependent decrease in MN induction by benzo[*a*]pyrene (Heo et al., 1992). Similarly, flavonol galangin showed the anti-mutagenic (Ames test) and anticlastogenic (MN test) capability against *N*-methyl-*N*-nitrosourea in mouse bone marrow cells and, therefore, may be a useful chemopreventive agent (Sohn et al., 1998). Free radical scavenging properties of naringin, a grapefruit flavanone, significantly reduced the amount of damages and MN in bleomycin-treated V79 cells (Jagetia et al., 2007). The other study by Choy et al. (1995) described the inhibition of urethane-induced MN by ethanol. Urethane is a mutagenic carcinogen that is found in fermented products and alcoholic beverages. Ethanol can inhibit certain pathways in the production of cytotoxic metabolites of urethane. The co-administration of urethane and ethanol in bone marrow polychromatic erythrocytes decreases MN frequency with an ethanol dose of 2500 mg/kg and above (Choy et al., 1995). A therapeutic drug cimetidine has been shown to protect bone marrow cells from radiation- and benzene-induced MN, most probably by free radical scavenging and the activation of the glutathione system along with cytochrome P450 inhibition (Mozdarani and Kamali, 1998). Another drug, fullerenol, decreased the frequency of MN in CHO-K1 cells due to its antioxidative properties (Mrdanovic et al., 2009). As expected, vitamin E prevents 30–50% of the toxic effect of zearalenone (non-steroidal mycotoxin), mainly by acting either as ZEN structural analog or as an antioxidant (Ouanes et al., 2003).

An interesting study showed an anticlastogenic effect of Ginkgo biloba extract (EGb 761) on patients with a hyperthyroid Grave’s disease. Such patients were treated with radioiodine therapy which causes chromosomal damages in the form of MN. The supplementation with EGb 761 during radioiodine treatment neutralized genotoxic effects without interfering with clinical outcomes (Dardano et al., 2007).

Finally, a surprisingly optimistic study described anti-carcinogenic effects of beer on aberrant crypt foci in the rat colon. Four commercial beers such as two Pilsner-type, black and stout showed inhibitory effects on heterocyclic amine-induced mutagenesis. Freeze-dried samples of pilsner and stout beer reduced the number of micronucleated cells, suggesting that beer inhibits genotoxic affects of heterocyclic amines and reduces the risk of carcinogenesis (Nozawa et al., 2004).

## THE FREQUENCY OF MICRONUCLEI FORMATION: LIFESTYLE FACTORS AND GENETIC POLYMORPHISMS

Different variants may modulate the effect of genotoxic agents on MN frequency. Mainly, they are host factors (age, gender), lifestyle (smoking, alcohol, occupation, folate, and vitamins intake), and disease susceptibility (cancer, etc.; Iarmarcovai et al., 2008a). Over the last century, multiple studies on MN formation have been described. The prospective analysis of such data shows the variability in MN frequency depending on the above-mentioned factors. Intra- and interindividual characteristics seem to account for MN formation. A comparison of three different mouse strains showed different sensitivity to the induction of MNPCEs by the same clastogenic effects (Sato et al., 1990).

Bolognesi et al. (1997) described an age-related increase in chromosome damages and MN formation in lymphocytes. Analysis of population data from 12 Italian laboratories in the mid 1980s–1990s showed the most dramatic increase in MN in the age group of 50–59 that remained unchanged thereafter (Bolognesi et al., 1997). The age-associated incline in CA and MN may be caused by a decline in DNA repair (Hanawalt et al., 1992) and the aneuploidy phenomenon (Richard et al., 1993). Genomic instability and oncogenicity cause the accumulation of DNA damage with age. Oxidative damage can also contribute to MN frequency during ageing (Ames and Shigenaga, 1992). The baseline MN frequency in newborns and children is relatively low, but higher susceptibility to DNA damages in children may rapidly increase the MN formation due to environmental exposure to genotoxic agents (Holland et al., 2011). Gender factors have been studied in parallel with aging. Mainly, a higher MN frequency has been reported for women (Barale et al., 1998). Similarly, the effect of gender was described for MN associated with aneuploidy (centromere-positive MN), which was higher in females (Iarmarcovai et al., 2007a). The frequency of X chromosome loss was also shown to be higher in females, especially in older women with X chromosome loss of approximately 22% (Bukvic et al., 2001). The impact of alcohol consumption on MN formation was also observed (Iarmarcovai et al., 2007a). The effect of smoking correlated linearly with chromosomal aberrations such as sister chromatid exchanges (Barale et al., 1998), and it surprisingly had no influence on MN formation (Bukvic et al., 2001).

Genetic polymorphisms have the major influence on interindividual susceptibility to MN formation (Iarmarcovai et al., 2007b). Single nucleotide polymorphisms in DNA repair genes XRCC1, XRCC3, and XPD (xeroderma pigmentosum group D) increased MN frequencies in radiological workers exposed to low levels of ionizing radiation compared to control individuals of the same genotype (Angelini et al., 2005). Similarly, glutathione *S*-transferase M1 polymorphisms influenced MN induction in coke oven workers, smokers, and subjects living in polluted areas (Sram, 1998). ALDH2 (aldehyde dehydrogenase 2) polymorphism is also associated with MN formation induced by alcohol (Ishikawa et al., 2003). For instance, ALDH2*2, an inactive variant allele of ALDH2, is highly present among Asian populations.

An individual predisposition to diseases, such as cancer, is correlated with MN incidence. MTHFR (methylenetetrahydrofolate reductase) variants involved in folate metabolism may develop into coronary artery disease (Andreassi et al., 2003). As mentioned previously, folate deficiency is often associated with an increase in the MN frequencies. Carriers of BRCA1 and BRCA2 mutations are predisposed to enhanced sensitivity to DNA damage, MN formation, and cancer development (Speit and Trenz, 2004).

Recently, van Leeuwen et al. (2011) developed a transcriptomic network analysis of MN-related genes based on the knowledge from literature and a case study on children and adults who were differentially exposed to air pollution. Using a pathway tool MetaCore, the authors retrieved 27 genes and gene complexes involved in MN formation. Such genes were mainly associated with cell cycle checkpoints, spindle assembly, and aneuploidy. The network was tested against a gene expression case study of individuals living in highly polluted mining area of Teplice (TP) in Czech Republic and less polluted area of Prachatice (PR) in the same country. Six genes from the network were retrieved in the exposed group where they were differentially expressed: BAX, PCNA, p21, CDC20, DNMT1, and HIC1. These genes were combined with p53 and IL-6 to create a micronucleus network (van Leeuwen et al., 2011). This study provided a novel, mechanistically relevant information on population response to genotoxic agents in relation to MN formation.

Further investigation may reveal novel genes which can be added to the network. Transcriptomic data may be incorporated in a future prediction and screening of genotoxic effects on MN, genomic instability, and cancer initiation.

## CONCLUSION

Due to their rapid formation and easy detection, MN have become the most prevalent biomarker of chromosomal defects induced by genotoxic agents. A variety of clastogenic and aneugenic toxins have been tested in different cells and tissues, using the MN test as one of the endpoints. The ability to distinguish between chromosome fragments and whole chromosomes lagging behind in anaphase allows the determination of the mechanisms of action of a variety of mutagenic agents. In the last decades, an enormous amount of data has shed light on the mode of MN formation, MN incidence in different cells and tissues among individuals, and the fate of MN in cells damaged by genotoxins. MNi are thought to reflect the initial stage in the development of genomic instability and tumorigenesis. Individuals predisposed to cancer tend to form MN more rapidly than persons without hereditary history. Therefore, MN screening may serve as a valuable method in predicting various diseases, including cancer. Cancer treatment therapies also rely on the ability to cause profound damage and induce death in malignant cells. The formation of MN in cancer cells undergoing new treatment indicates the activity and specificity of the tested therapy. On the other hand, common drugs may cause side effects if the MN induction in non-target cells is observed. Biomonitoring studies aimed to understand the effect of environment on populations with different lifestyles and sensitivity to genotoxins are of a great value. The transcriptome network reflecting genes associated with MN formation may help to better understand the value of chromosome damages induced by surrounding genotoxins.

Although MN studies are the leading field in mutation research, some important knowledge gaps remain uncovered. Namely, it is not fully addressed whether MN re-engulfed by the cell can restore a complete genotype in the cell, and whether MN can contribute to cellular gene expression. The exact mechanism of MN content degradation is also under question. Better understanding of gene interactions responsible for different MN frequencies would help to improve drug testing, biomonitoring of diseases and the prediction of the effects of common genotoxic agents. Moreover, more studies are needed to understand the role of epigenetic factors in MN formation.

## Conflict of Interest Statement

The authors declare that the research was conducted in the absence of any commercial or financial relationships that could be construed as a potential conflict of interest.
